# Miscellanea

**Published:** 1856-10

**Authors:** 


### MISCELLANEA.

### A NEW GAS STOVE.

Mr. Goode, of Birmingham, has forwarded to us a description of two new gas-stoves, which he has recently invented. One is intended to act as a cooking-stove, the other as a heating and ventilating stove. In both stoves, the flame and heated air, from a central burner, strike against the bottom of an inner chamber, and communicate their heat to it. In the cooking-stove the food to be cooked is placed in this chamber, and is roasted or heated out of contact with the vitiated air, in which combustion has taken place. In the heating and ventilating stove, a current of pure and warm air is delivered into the apartment, uncontaminated with the foul air from the burner. The air in which combustion has taken place is conducted by a flue from the apartment. Air from the room is introduced into the inner chamber by tubes arranged round the burner. The advantages which Mr. Goode believes to be obtained in this stove are, that the maximum amount of heat is obtained from the burning gas, while the air of the room is maintained in a state of absolute purity.

These stoves are also constructed very cheap, occupy but little space, and supply the heat at a small cost, as far as the consumption of gas is concerned. The stoves certainly rectify many of the faults of ordinary gas-stoves, the admission into the room of air containing the products of the combustion of the gas being very effectually prevented.

